# Exploring the Regulatory Mechanism of Sodium Butyrate on Milk Fat Synthesis in Lactating Goats Fed a High-Concentrate Diet Based on Metabolomics Technology

**DOI:** 10.33549/physiolres.935816

**Published:** 2026-08-01

**Authors:** Lin LI, Yanfen LYU, Hongxia HOU, Binbin GONG, Dingxiang LIU, Ruiai CHEN

**Affiliations:** 1 College of Veterinary Medicine, South China Agricultural University, Guangzhou, China; 2 Department of Food Science and Bioengineering, Xingtai University, Xingtai, China; 3 Zhaoqing Branch Center of Guangdong Laboratory for Lingnan Modern Agricultural Science and Technology, Zhaoqing, China; 4 School of Mathematics and Physics, Hebei University of Engineering, Handan, China

**Keywords:** Metabolomics, Sodium butyrate, Mammary gland, Milk fat, Metabolism regulation

## Abstract

This study investigated the effects of sodium butyrate (SB) supplementation on milk fat synthesis in lactating goats fed a high-concentrate (HC) diet. Twelve lactating Saanen goats were randomly allocated into two groups: a control group (HG) fed a HC diet (concentrate-to-forage ratio = 60:40), and a SB-treated group (SG) fed the same basal diet supplemented with 10 g/kg SB. Compared with the HG group, SB supplementation significantly increased milk fat percentage and milk fat yield (P < 0.01). Meanwhile, SB significantly decreased lipopolysaccharide (LPS) concentrations in both jugular and mammary vein plasma (P < 0.05). In addition, SB enhanced mammary antioxidant capacity, as indicated by elevated activities of superoxide dismutase (SOD) (P < 0.01) and total antioxidant capacity (T-AOC) (P < 0.05). Global metabolomic analysis (UPLC-MS/MS) of mammary gland tissues identified 558 differential metabolites, of which 50 were significantly altered and mainly enriched in amino acids, benzene derivatives, heterocyclic compounds, and fatty acids. Pathway analysis showed that SB significantly activated the pentose phosphate pathway, glycerophospholipid metabolism, and linolenic acid metabolism. Furthermore, mammary gland triglyceride (TG) content was significantly higher in the SG group than in the HG group (P < 0.05). SB also upregulated the mRNA expression of key lipogenic enzymes, including acetyl-CoA carboxylase (ACC) and fatty acid synthase (FAS) (P < 0.05). These findings demonstrate that SB improves mammary gland health and mitigates the adverse effects of HC diets on milk quality by enhancing de novo fatty acid synthesis in the mammary gland of lactating goats.

## Introduction

To meet the high-energy demands of ruminants during lactation, high-concentrate (HC) diets are commonly supplemented to support milk production in animals such as cows and goats [1,2]. However, long-term HC feeding may induce subacute ruminal acidosis, leading to the excessive production of lipopolysaccharide (LPS) in the rumen [3]. LPS, also known as endotoxin, is a component released from the cell walls of Gram-negative bacteria upon lysis. It can enter the bloodstream and disseminate to various organs, triggering systemic inflammation and contributing to conditions such as liver injury and mastitis [4–7]. Furthermore, HC feeding in lactating dairy cows may result in reduced milk fat content and overall milk yield. This is partly attributed to the suppression of key enzyme activities involved in mammary gland fatty acid synthesis, thereby impairing milk fat production [8]. The adverse effects associated with HC diets have significantly hampered the sustainable development of the dairy industry, highlighting the critical need for effective strategies to mitigate these negative impacts.

Sodium butyrate (SB), whose primary active component is butyric acid, can permeate cell membranes to inhibit the growth of harmful bacterial communities, thereby promoting the proliferation of beneficial gut microbiota and maintaining a healthy balance in the gastrointestinal microbiome [9]. SB also plays a significant role in exerting anti-inflammatory and tissue-protective effects in various organs of ruminants, and suppresses LPS-induced inflammation in bovine mammary epithelial cells through the NF-κB pathway [10]. Supplementation with SB effectively mitigated LPS-induced oxidative stress in bovine mammary epithelial cells, thereby preventing cell death [11]. In addition, it modulates metabolic processes and enhances immune function [12,13]. Based on its multifaceted functions, we investigated the effects of an SB-supplemented HC diet on the metabolic expression profile in the mammary gland of lactating goats.

Untargeted metabolomics is a widely used approach in metabolomic research. It involves the comprehensive measurement and quantification of metabolites within biological samples to identify differentially abundant metabolites with statistical significance between comparative groups. The findings are then utilized to interpret the associations between the discovered metabolites and specific biological processes or states, thereby providing a foundation for elucidating the metabolic pathways and regulatory mechanisms in living organisms. Therefore, this study employed metabolomics to delineate the specific effects of SB-supplemented HC diet on mammary gland metabolites in lactating goats induced by a HC diet. The objective was to clarify the metabolic relationship with milk quality and explore a viable approach for its improvement.

## Materials and Methods

### Animals and experimental design

Twelve healthy Saanen dairy goats (40.12 ± 5 kg, Mean ± SEM; 3–4 weeks postpartum) with similar body weight and lactation stage were selected for this study. They were housed in individual cages at the Experimental Animal Centre (Nanjing Agricultural University, China). All goats were fed a HC diet for one week as an adaptation period to achieve a comparable metabolic state. All goats were randomly divided into two groups: one group received a HC diet (Concentrate: Forage = 60:40, HG, n=6) and the other group received the same basal diet supplemented with SB (Sigma, St. Louis, MO, USA) at 10 g/kg basal diet (SG, n=6). The composition and nutritional profile of the basal diets are presented in Table 1. The goats were fed a basal diet (1 kg dry matter per day) twice daily at 08:00 and 18:00 h. Milking was performed concurrently with feeding, and the daily milk yield was recorded. Throughout the 20-week experimental period, the animals had free access to water. During the 20^th^ week of the trial, milk samples were collected and analyzed for milk composition. The analysis was performed with an Integrated Milk-Testing™ Milkoscan 4000 analyzer (Foss Electric, Hillerød, Denmark) at the Weigang Dairy Testing Center in Nanjing, Jiangsu Province, China.

### Sample collection

At the end of the experiment, each goat was anaesthetized with 0.5 mL of xylazine hydrochloride. Blood samples were collected via the jugular vein and mammary vein, followed by centrifugation at 3000 × g for 15 min. The plasma was harvested and stored at −20 °C for subsequent LPS detection.

All the goats were slaughtered after blood samples were collected, and mammary tissues were then excised, segmented into small pieces, placed into cryovials, and snap-frozen at −80 °C for storage.

### LPS measurements

The LPS concentrations in jugular and mammary vein plasma samples were measured using a chromogenic endpoint assay (Chinese Horseshoe Crab Reagent Manufactory, Co., Ltd., Xiamen, China) following the manufacturer’s instructions.

### Determination of mammary gland oxidative stress indicators and triglyceride (TG) content

The activities of key antioxidant enzymes, including glutathione peroxidase (GSH-Px), superoxide dismutase (SOD), catalase (CAT), total antioxidant capacity (T-AOC), as well as the oxidative stress marker malondialdehyde (MDA), were assayed in mammary gland tissue using commercial assay kits. All commercial kits were purchased from the Nanjing Jiancheng Bioengineering Institute (Nanjing, China).

TG content was detected using the GPO-PAP method with a triglyceride detection kit, and the operation steps were performed in accordance with the kit instructions.

#### Mammary gland metabolomics

##### Mammary gland metabolite extraction

Frozen tissue samples were retrieved from −80 °C storage and thawed on ice (all subsequent procedures were performed on ice). The samples were uniformly powdered under liquid nitrogen conditions, and a 20 mg (±1 mg) aliquot of the homogenized powder was accurately weighed into correspondingly labeled centrifuge tubes. Subsequently, 400 μL of a 70 % methanol aqueous solution containing internal standards was added to each tube. The mixtures were vortexed at 1500 rpm for 5 min and then incubated on ice for 15 min. Following incubation, the tubes were centrifuged at 12,000 rpm for 10 min at 4 °C. A 300 μL aliquot of the resulting supernatant was carefully transferred to a new set of labeled tubes and maintained at −20 °C for 30 min. The extracts were then centrifuged again under the same conditions (12,000 rpm, 4 °C) for 3 min. Finally, 200 μL of the clear supernatant was transferred to a vial insert for instrumental analysis.

##### UPLC–MS/MS analysis

The LC analysis was conducted using a Waters ACQUITY Premier HSS T3 Column (1.8 μm, 2.1 mm × 100 mm). The mobile phase system comprised 0.1 % (v/v) formic acid in water (A) and 0.1% (v/v) formic acid in acetonitrile (B). The separation was carried out at a constant column temperature of 40 °C. A flow rate of 0.4 mL/min and an injection volume of 4 μL were employed for the analysis (Table 2).

##### Data pre-processing

The raw mass spectrometry data were converted to mzXML format using ProteoWizard software. Peak detection, alignment, and retention time correction were then performed with the XCMS package. Metabolic features with a missing rate exceeding 50 % across all samples were filtered out. Subsequently, the remaining missing values were imputed using the k-nearest neighbors (KNN) algorithm, and signal drift was corrected by the support vector regression (SVR) method. Metabolite identification was carried out by querying processed features against a custom laboratory-built database, integrated public databases, *in silico* prediction libraries, and utilizing the metDNA algorithm. Finally, a high-confidence dataset was constructed by retaining metabolites with an identification score above 0.5 and a coefficient of variation (CV) below 0.3 in quality control (QC) samples. Ions from positive and negative ionization modes were merged, retaining the annotation with the highest identification confidence and the lowest CV, resulting in the final “ALL sample_data” file.

##### RNA isolation, cDNA synthesis, and real-time PCR

Total RNA was extracted from mammary gland samples with TRIzol reagent (15596026, Invitrogen, Shanghai, China) following the manufacturer’s instructions. Subsequently, cDNA was synthesized from the extracted RNA using a commercial reverse transcription kit (Vazyme, Nanjing, China). Quantitative real-time PCR (qPCR) was performed using the AceQ qPCR SYBR Green Master Mix (Vazyme, Nanjing, China) on a MyiQ2 Real-Time PCR System (Bio-Rad, USA). The amplification protocol consisted of an initial denaturation at 95 °C for 2 min, followed by 40 cycles of 95 °C for 15 sec and 58–60 °C for 30 sec. All primer sequences used are listed in Table 3; they were synthesized by Generay Biotech (Shanghai, China). The relative mRNA expression levels were calculated using the 2^−ΔΔCt^ method, with glyceraldehyde 3-phosphate dehydrogenase (GAPDH) as the endogenous control for normalization, and are presented as fold changes relative to the mean value of the HG group.

##### Statistical analysis

All data are presented as the means ± standard error of the mean (SEM). The normality of data distribution was tested using the Shapiro-Wilk test, and homogeneity of variances was verified by Levene’s test. Differences between the HG and SG groups were analyzed by independent-samples T-test using SPSS 20.0 software (SPSS Inc., Chicago, IL, USA). *P < 0.05 indicated a significant difference, **P < 0.01 indicated an extremely significant difference, and P > 0.05 indicated no significant difference. For metabolomic data, differential metabolites were screened with VIP > 1, P < 0.05, and FC ≥ 2 or ≤ 0.5. Correlation analysis was performed using Pearson’s correlation coefficient.

## Results

### Effect of SB on the milk composition of lactating goats fed a HC diet

Compared with the HG group, SB supplementation extremely significantly increased milk fat percentage by 20.4 % and milk fat yield by 23.4 % (P < 0.01), without affecting milk yield, milk protein, or lactose (P > 0.05) (Table 4).

### Measurement of LPS content in blood of lactating goats

**Fig. 1:**
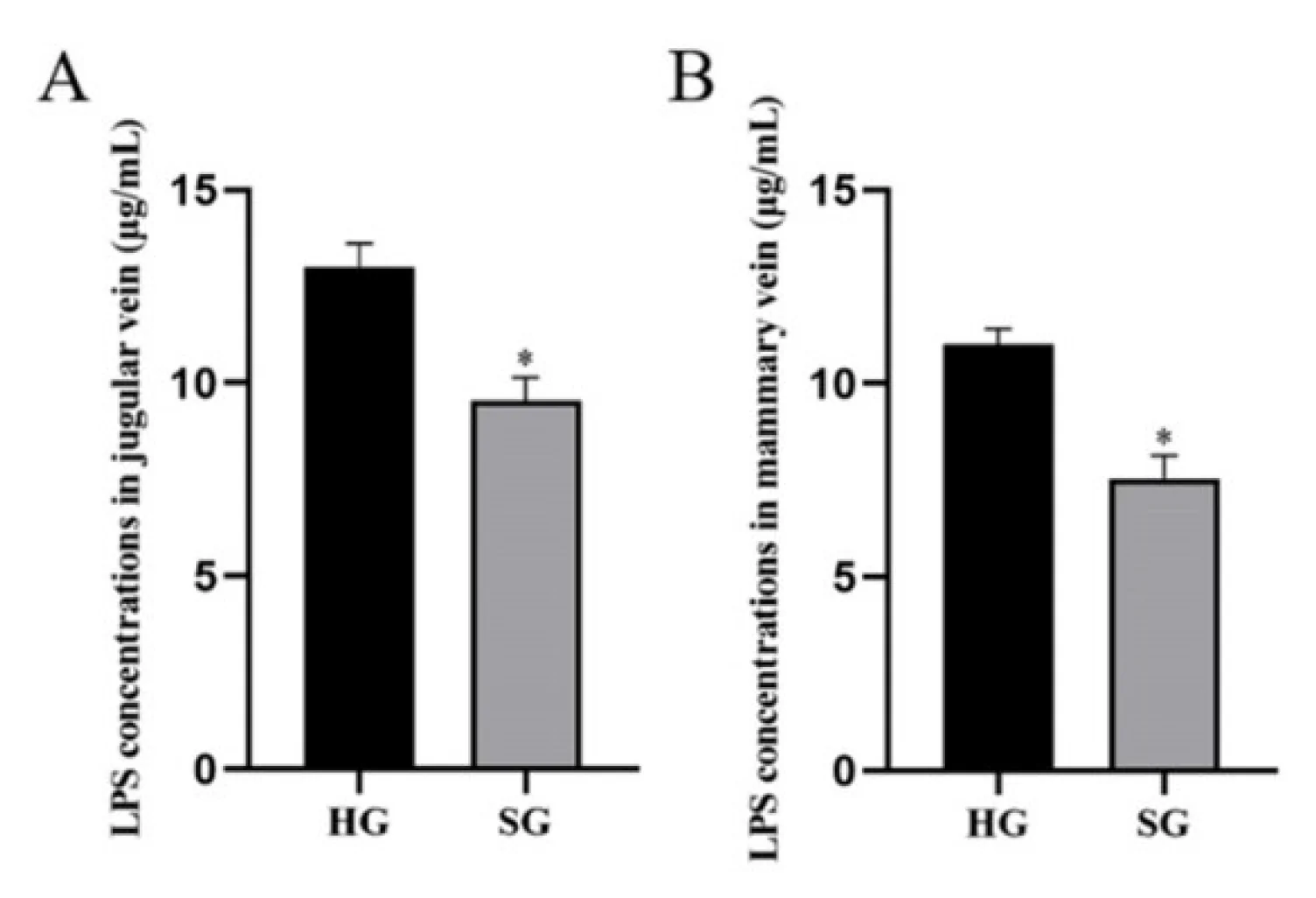
Effect of sodium butyrate on LPS content in the blood of lactating goats. Data were analyzed with independent sample T-tests and expressed as mean ± SEM (n=6 per group). *P < 0.05 vs. HG group.

The LPS content in the blood of the two groups of lactating goats was detected. As shown in the Fig. 1, the LPS content in the jugular and mammary venous blood of the SG group was significantly lower than that in the HG group (P < 0.05), indicating that SB supplementation reduced the generation of the abnormal product, LPS.

### Measurement of oxidative stress indices in the mammary gland of lactating goats

As shown in Table 5, mammary gland antioxidant indices including SOD, CAT, and T-AOC were higher in the SG group than in the HG group. Specifically, the increases in SOD (P < 0.01) and T-AOC (P < 0.05) were statistically significant. These results indicate that SB supplementation alleviated the mammary gland oxidative stress induced by the HC diet in lactating goats.

### Analysis of the mammary gland metabolism patterns in lactating goats fed a HC diet with SB

**Fig. 2:**
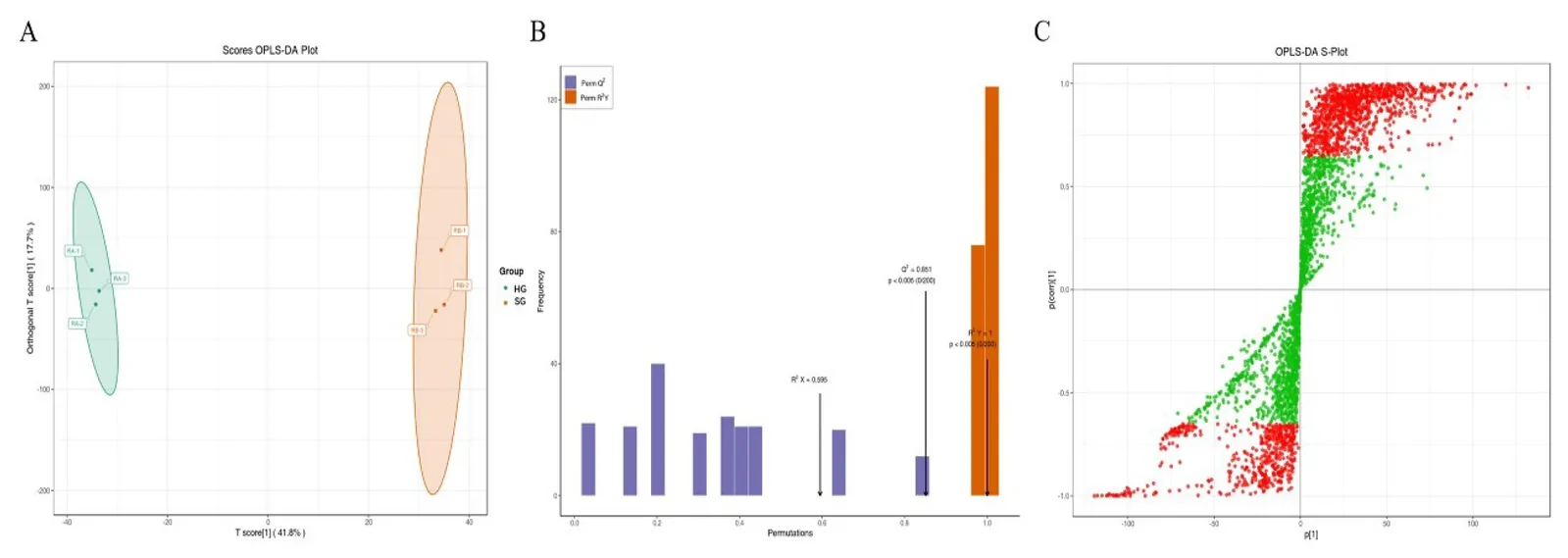
OPLS-DA score plots, validation plots, and S-plots between the two groups of goats. **(A)** OPLS-DA score plots. The x-axis represents the score values of the predictive component; the y-axis represents the score values of the orthogonal component; the percentages indicate the explained variance of each component in the dataset. **(B)** OPLS-DA validation plots. The x-axis displays the R^2^Y and Q^2^ values of the model, while the y-axis represents the frequency of the model’s classification performance observed in 200 permutation tests. The orange bars correspond to the R^2^Y values of the permuted models, the purple bars represent the Q^2^ values of the permuted models, and the black arrows indicate the R^2^X, R^2^Y, and Q^2^ values of the original model. **(C)** OPLS-DA S-plots. The x-axis represents the covariance between the principal component and metabolites, while the y-axis indicates the correlation coefficient between the principal component and metabolites. The red points denote metabolites with VIP values greater than 1, and the green points represent those with VIP values less than or equal to 1.

Orthogonal Projections to Latent Structures-Discriminant Analysis (OPLS-DA) was employed to discriminate the metabolic profiles between the two groups of lactating goats. The resulting model, visualized in the score plot (Fig. 2A), demonstrated a clear separation between groups and a tight clustering of samples within each group, with no obvious outliers. This indicates a distinct metabolic difference between the two cohorts. The model’s robustness was validated by a 200-permutation test, which yielded high values for the explained variance (R^2^X = 0.595, R^2^Y = 1) and predictive ability (Q^2^ = 0.851). In OPLS-DA, R^2^X and R^2^Y represent the fraction of variance explained in the X (variables) and Y (response) matrices, respectively, while Q^2^ denotes the predictive capability of the model. A model is generally considered reliable when these parameters approach 1, with Q^2^ > 0.5 indicating a valid model and Q^2^ > 0.9 representing an excellent one (Fig. 2B). The S-plot (Fig. 2C) was used to identify significant metabolites contributing to the separation. The x-axis represents the covariance, and the y-axis the correlation, between metabolites and the predictive component. Metabolites located in the far right or left corners of the “S” are the most significant. Those highlighted in red have a Variable Importance in Projection (VIP) score > 1, while those in green have a VIP ≤ 1. Collectively, these data confirm that the OPLS-DA model constructed in this study possesses strong predictive power and is suitable for the subsequent screening of differential metabolites.

### Screening of differentially abundant metabolites in the mammary gland

**Fig. 3:**
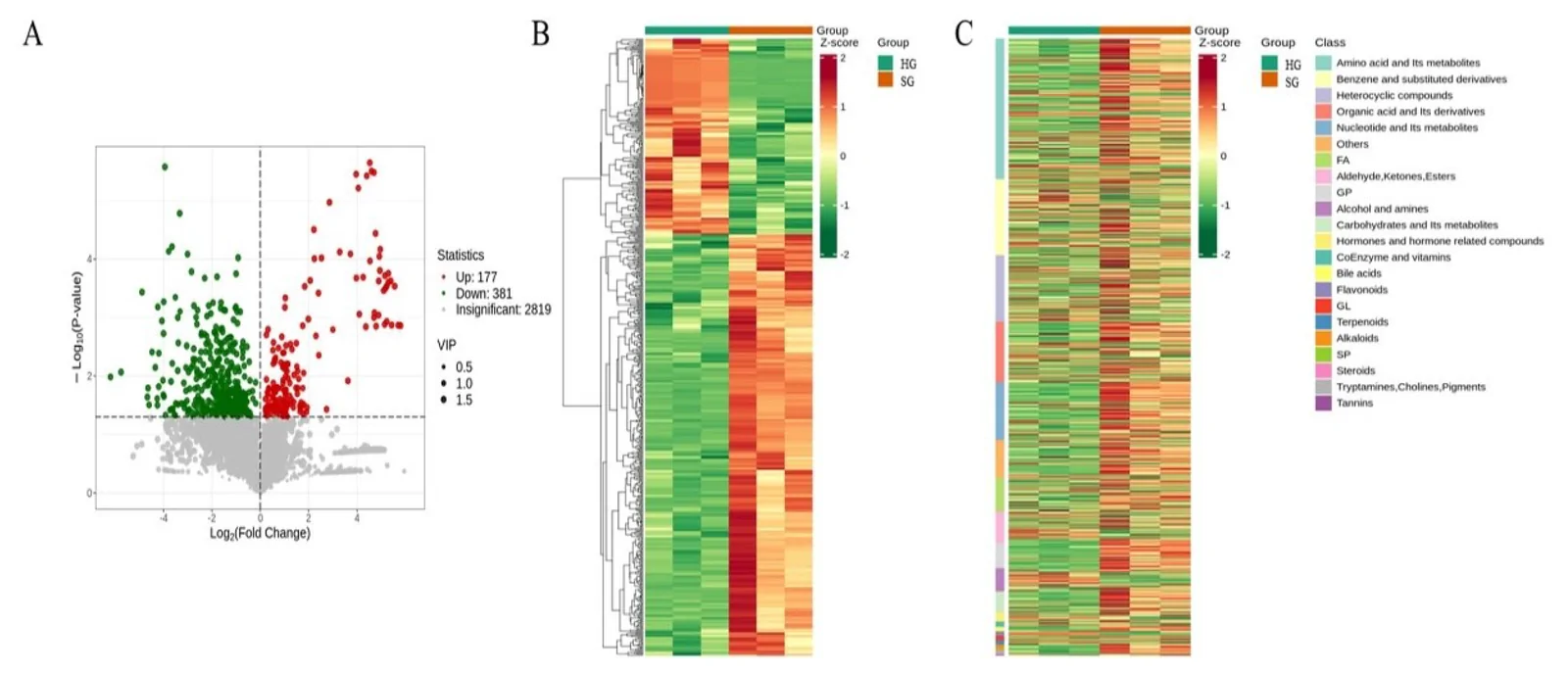
Analysis of differentially abundant metabolites in the two groups of goats. **(A)** Metabolite volcano plot. The green points represent the down-regulated differential metabolites, and the red points represent the up-regulated ones. **(B**–**C)** Heat map of differential metabolite clustering. The rows represent the differential metabolites, and the columns represent the individual samples. The annotation “Group” indicates the sample designation, with the color scale ranging from red (indicating highly abundant metabolites) to green (indicating less abundant metabolites).

Based on the OPLS-DA model, potential differential metabolites were screened by applying the following criteria: a Variable Importance in Projection (VIP) value greater than 1, a p-value less than 0.05, and a fold change (FC) of either ≥ 2 (up-regulated) or ≤ 0.5 (down-regulated). Compared to the HG group, a total of 558 differential metabolites were identified in the SG groups, among which 177 were significantly up-regulated and 381 were down-regulated (Fig. 3A). The heatmap in Fig. 3B shows the result of a clustering analysis conducted on the differential metabolites. Clear and distinct clustering of mammary gland samples from the HG and SG group was observed, indicating substantial differences in mammary gland metabolism.

To investigate the patterns of metabolite abundance, the raw relative abundances of the identified differential metabolites were normalized using Unit Variance scaling. Subsequently, we found that these metabolites were predominantly enriched in several classes, including Amino acid and its metabolites, Benzene and substituted derivatives, Heterocyclic compounds, Organic acid and Its derivatives, Nucleotide and Its metabolites, fatty acid (FA) and other metabolites, compared to the HG group (Fig. 3C).

### Pathway analysis of differentially abundant mammary gland metabolites

**Fig. 4:**
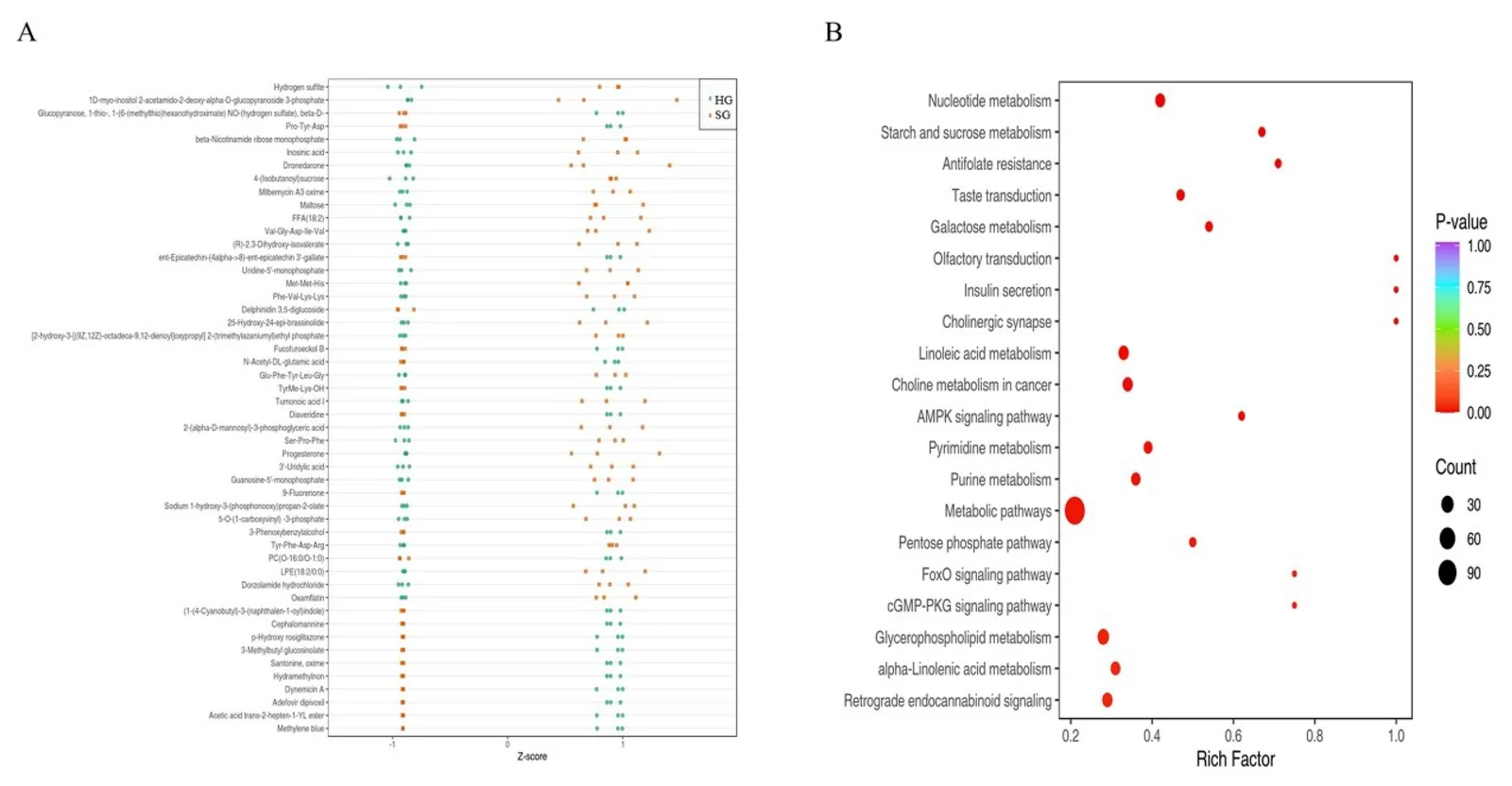
Analysis of differential metabolites and KEGG pathways in the two groups of goats. **(A)** Z-score heatmap of differential metabolites. In the plot, the x-axis represents the Z-score, the y-axis lists the metabolites, and the colored points correspond to samples from different groups. **(B)** KEGG enrichment plot of differential metabolites. The x-axis represents the Rich Factor for each pathway, and the y-axis displays the pathway names (sorted by P-value). The color of the dots corresponds to the P-value, with a darker red color indicating a more significant enrichment.

Pearson correlation coefficients were calculated to assess the correlations among the top 50 differentially abundant metabolites. Notably, the abundances of three metabolites-FFA (18:2), LPE(18:2/0:0), and [2-hydroxy-3-[(9Z,12Z)-octadeca-9,12-dienoyl]oxypropyl] 2-(trimethyla-zaniumyl)ethyl phosphate-were significantly elevated in the SG group relative to the HG group (Table 6 and Fig. 4A). Furthermore, all the pathways of the differential metabolite mapped were sorted out through thedatabase. The pathway impact analysis suggested that SB produced a significant impact on 20 metabolic pathways, including Nucleotide metabolism, Starch and sucrose metabolism, Antifolate resistance, Taste transduction, Galactose metabolism, Cholinergic synapse, Insulin secretion, Olfactory transduction, Linoleic acid metabolism, Choline metabolism in cancer, AMPK signaling pathway, Pyrimidine metabolism, Purine metabolism, Metabolic pathways, Pentose phosphate pathway, FoxO signaling pathway, cGMP-PKG signaling pathway, Glycerophospho-lipid metabolism, alpha-Linolenic acid metabolism, Retrograde endocannabinoid signaling, as shown in Fig. 4B.

### Effect of SB on TG content in the mammary gland of lactating goats

**Fig. 5:**
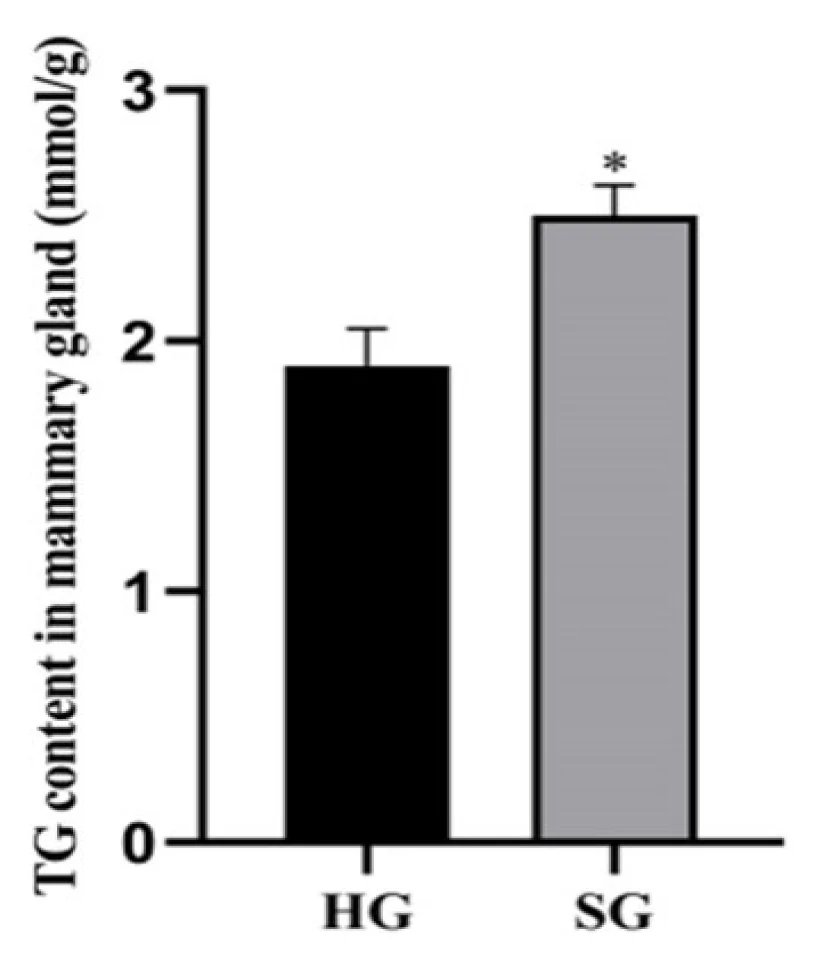
TG contents in the mammary gland of lactating goats. Data were analyzed with independent sample T-tests and expressed as mean ± SEM (n=6 per group). *P < 0.05 vs. HG group.

The results showed that the TG content in the mammary gland of the SG group was significantly higher than that in the HG group (P < 0.05) (Fig. 5). These findings indicate that SB promotes the production of more milk fat precursors (TG), which are subsequently utilized for milk fat synthesis.

### SB treatment regulated key enzymes involved in lipid metabolism in the mammary gland of goats

**Fig. 6:**
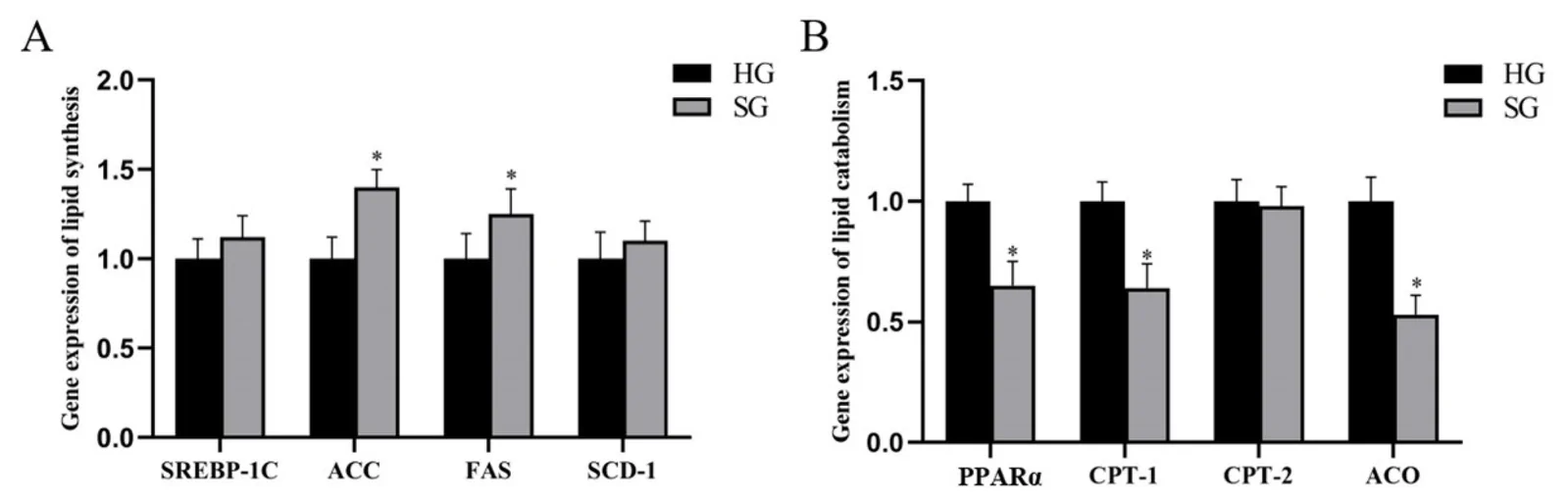
Effects of sodium butyrate on the expression of mammary gland lipid metabolism in lactating goats. Data were analyzed with independent sample T-tests and expressed as mean ± SEM (n=6 per group). *P < 0.05 vs. HG group.

The SB diet increased the expression of SREBP-1c downstream targets, which included ACC, FAS, and SCD-1. The expression of ACC and FAS was significantly higher in the SB group than in the HG goats (P < 0.05, Fig. 6A). Compared with the HG group, the SG group presented significantly lower PPARα, CPT-1 and ACO expression levels (P < 0.05; Fig. 6B). In contrast, CPT-2 expression did not differ significantly between the SG and HG groups (P > 0.05).

## Discussion

Milk quality is influenced by a multitude of factors, including breed, parity, dietary forage-to-concentrate ratio, and environmental conditions [14]. In the dairy industry, feeding HC diets to lactating animals is a widely adopted practice to enhance milk yield and overall cost-effectiveness. However, this feeding strategy also promotes rapid acid production, increasing LPS levels and facilitating its translocation into the bloodstream, ultimately compromising milk quality and animal health [15,16]. Long-term consumption of a HC diet promotes the translocation of LPS from the digestive tract into the systemic circulation, particularly the peripheral circulatory system, thereby triggering a systemic inflammatory response. Research has found that a high-concentrate diet leads to a significant elevation of LPS in the bloodstream and induces impaired mitochondrial function in dairy cows by activating the MAPK signaling pathway in the mammary gland [17]. Similarly, LPS induces oxidative stress by triggering MAPK and Nrf2 signalling pathways in mammary glands of dairy cows fed a HC diet [18].

Butyric acid (C4), a short-chain fatty acid, can be oxidized to generate energy in various tissues. As a crucial energy source for the gastrointestinal tract, it suppresses pathogenic bacteria and helps maintain gut homeostasis. Aguilar *et al*. reported that butyrate alleviates inflammation through multiple mechanisms, including reduced immune cell infiltration, promotion of anti-inflammatory cytokine secretion, and enhanced antioxidant production [19]. Furthermore, studies have shown that SB exhibits multi-target antioxidant activity, protecting tissues from free radical damage [20,21]. In an experimental study, Memon *et al*. demonstrated that SB alleviates high-concentrate diet-induced oxidative stress in the mammary gland of lactating goats by upregulating catalase expression and suppressing superoxide anion generation [22]. Our findings demonstrate that SB not only reduced plasma LPS levels but also significantly increased the activities of the antioxidant enzymes SOD and T-AOC in mammary tissue. These results suggest a dual protective mechanism: SB may improve systemic health in lactating goats by attenuating circulating LPS, while concurrently enhancing the antioxidant defense in mammary cells, thereby reducing intracellular oxidative damage.

SB, whose active component is butyric acid, is produced by gut microbiota through the fermentation of non-digestible carbohydrates such as fibre. As a feed additive, it has been shown to enhance growth performance, reduce the incidence of mastitis, and increase milk fat content in livestock [23,24]. Previous studies indicate that long-term intake of HC diets can induce metabolic disorders in goats, leading to a reduction in milk fat [25]. It has been reported that dietary supplementation with sodium butyrate helps regulate metabolism and improve lactation performance [26]. In the present study, we observed that goats supplemented with SB exhibited higher milk fat percentage and total milk fat yield compared with the HG group. These findings indicate that SB holds promising potential for improving milk quality, particularly in ruminants fed HC diets. Nevertheless, the underlying mechanism through which SB modulates milk fat synthesis warrants further investigation.

In the mammary gland, milk fat synthesis is a coordinated process involving de novo fatty acid synthesis, fatty acid uptake, triglyceride formation, and lipid droplet secretion. We employed metabolomics to investigate the effects of SB on the mammary gland metabolome. Comparative analysis between the two groups revealed differential accumulation of multiple metabolites. Notably, the SB-supplemented group showed significantly higher levels of linoleic acid (FFA 18:2), LPE(18:2/0:0), and a choline-containing phospholipid identified as [2-hydroxy-3- [(9Z,12Z) -octadeca-9,12-die- noyl]oxypropyl]2- (trimethylazaniumyl)ethyl phosphate. These metabolites are important intermediates or components involved in glycerophospholipid metabolism and linoleic acid metabolism, which provide critical structural lipids, signaling molecules, and synthetic precursors for milk fat formation in the mammary gland.

According to KEGG database analysis, our metabolomic results revealed that SB significantly enriched glycerophospholipid metabolism, linoleic acid metabolism, the pentose phosphate pathway, and the AMPK signaling pathway. Notably, the mammary gland is one of the tissues with the highest pentose phosphate pathway activity in the body, whose primary function is to generate abundant Nicotinamide adenine dinucleotide phosphate (NADPH) that provides the essential reducing power for de novo milk fat synthesis [27]. Collectively, these pathways synergistically provide critical precursors (from glycerophospholipid metabolism and linoleic acid metabolism), reducing power (from the pentose phosphate pathway), and regulatory signals (from the AMPK signaling pathway) to support mammary lipid anabolism, which is consistent with the key role of the mammary gland in milk fat synthesis.

Milk fat, an essential nutritional component of milk, is widely recognized for its health benefits. However, prolonged consumption of a HC diet has been shown to reduce milk fat content [28]. TG, the primary constituents of milk fat, are synthesized from fatty acids and α-glycerophosphate within mammary epithelial cells. Finally, through a series of packaging and processing steps, TG is released into the mammary alveoli to form milk fat [29]. Sterol regulatory element-binding proteins (SREBPs) are key transcription factors regulating lipid and fatty acid synthesis, among which SREBP-1c controls the expression of genes related to lipid synthesis and deposition, such as ACC, SCD-1, and FAS [30]. In addition, PPARα plays an essential role in regulating mitochondrial and peroxisomal fatty acid oxidation in ruminants and modulates downstream targets including CPT-1, CPT-2, and ACO [31]. Previous studies have indicated that SB enhances milk fat synthesis in bovine mammary epithelial cells via the GPR41-mediated signaling pathway [32].

In the present study, we found that at the transcriptional level, SB significantly upregulated the expression of ACC and FAS, which are the core regulators and rate-limiting enzymes controlling de novo fatty acid synthesis in mammary epithelial cells. Meanwhile, SB downregulated the expression of PPARα, CPT-1, and ACO, which are the key genes promoting mitochondrial fatty acid oxidation. These changes indicated that SB enhanced lipid anabolism and reduced lipid catabolism in the mammary gland. Consistent with these regulations, mammary gland TG content was significantly increased, which directly contributed to the higher milk fat percentage and yield. Taken together, SB promotes milk fat synthesis by systematically regulating the lipid synthesis pathway in the mammary gland, including enhancing metabolic precursor supply, activating key lipogenic gene expression, and inhibiting fatty acid degradation.

## Conclusions

This study demonstrated that SB supplementation improved mammary gland health and increased milk fat content in HC-fed lactating goats. SB reduced plasma LPS levels, enhanced mammary antioxidant capacity. Metabolomic analysis revealed that SB orchestrated key metabolic pathways in the mammary gland, including the pentose phosphate pathway, glycerophospholipid metabolism and linoleic acid metabolism, thereby supplying adequate precursors for milk fat synthesis. These findings indicate that SB promotes milk fat production by enhancing mammary health, optimizing lipid metabolic profiles, and stimulating lipid anabolism in goats fed a high-concentrate diet.

## Figures and Tables

**Table 1:** Composition and nutrient levels of the basal diets.

Concentrate: Forage ratio 60:40			
Ingredient (% of dry matter)		Nutrient level^b^	
*Leymus chinensis*	27.00	Net energy/(MJ.kg^−1^)	6.71
*Alfalfa silage*	13.00	Crude protein/%	16.92
*Corn*	23.24	Neutral detergent fibre/%	31.45
*Wheat bran*	20.77	Acid detergent fibre/%	17.56
*Soybean meal*	13.67	Calcium/%	0.89
*Limestone*	1.42	Phosphorus/%	0.46
*NaCl*	0.30		
*Premix* a	0.60		
*Total*	100.00		

a - Guaranteed levels provided per kg of diet: vitamin A: 6.000 IU/kg (min.); vitamin D: 2.500 IU/kg (min.); vitamin E: 20.000 IU/kg (min.); Cu: 6.25 mg/kg (min.); Fe: 62.5 mg/kg (min.); Zn: 62.5 mg/kg (min.); Mn: 50 mg/kg (min.); I: 0.125 mg/kg (min.); Co: 0.125 mg/kg (min.).

b - Nutrient levels were estimated values.

**Table 2:** Gradient Elution Program for the T3 Column.

Time (min)	A (%)	B (%)
0.0	95	5
2.0	80	20
5.0	40	60
6.0	1	99
7.5	1	99
7.6	95	5
10.0	95	5

**Table 3:** Primer sequences used for qRT-PCR analysis of target genes in lactating goats.

Target genes	Primer sequences (5′-3′)	Products/bp
*GAPDH*	GGGTCATCATCTCTGCACCT GGTCATAAGTCCCTCCACGA	177
*SREBP-1c*	CGACTACATCCGCTTCCTTCA ACTTCCACCGCTGCTACTG	259
*ACC*	ACGCAGGCATCAGAAGATTA GAGGGTTCAGTTCCAGAAAGTA	179
*FAS*	GCACTACCACAACCCAAACCC CGTTGGAGCCACCGAAGC	161
*SCD-1*	CCGCCCTGAAATGAGAGATG AGGGCTCCCAAGTGTAACAGAC	154
*PPARα*	GGAGGTCCGCATCTTCCACT GCAGCAAATGATAGCAGCCACA	352
*CPT-1*	CCCATGTCCTTGTAATGAGCCAG AGACTTCGCTGAGCAGTGCCA	230
*CPT-2*	ACGCCGTGAAGTATAACCCT CCAAAAATCGCTTGTCCCTT	119
*ACO*	TAAGCCTTTGCCAGGTATT ATGGTCCCGTAGGTCAG	189

GAPDH: glyceraldehyde 3-phosphate dehydrogenase; SREBP-1c: sterol regulatory element-binding protein-1c; ACC: acetyl-CoA carboxylase; FAS: fatty acid synthetase; SCD-1: stearoyl-CoA desaturase 1; PPARα: peroxisome proliferator-activated receptor α CPT-1: carnitine palmitoyl transferase-1; CPT-2: carnitine palmitoyl transferase-2; ACO: acyl-CoA oxidase.

**Table 4:** Effect of sodium butyrate on the performance of lactating goats.

Item	HG	SG	P-value
*Milk yield, g/d*	975.34 ± 23.12	998.36 ± 54.45	>0.05
*Milk protein, %*	2.94 ± 0.21	3.04 ± 0.12	>0.05
*Milk protein, g/d*	30.21 ± 1.47	31.12 ± 1.14	>0.05
*Milk fat, %*	3.13 ± 0.12	3.77 ± 0.12**	<0.01
*Milk fat, g/d*	32.14 ± 0.45	39.67 ± 0.66**	<0.01
*Lactose, %*	3.57 ± 0.32	3.62 ± 0.29	>0.05
*Lactose, g/d*	35.14 ± 2.36	36.38 ± 3.21	>0.05

Data were analyzed with independent sample T-tests and expressed as mean ± SEM (n=6 per group).

** P < 0.01 vs. HG group.

**Table 5:** Effect of sodium butyrate on mammary gland oxidative stress index of lactating goats.

Item	HG	SG	P-value
*GSH-Px, U/gprot*	25.12 ± 2.36	24.23 ± 3.26	>0.05
*SOD, U/mgprot*	16.11 ± 1.13	24.54 ± 0.88**	<0.01
*CAT, U/mgprot*	2.63 ± 0.25	2.85 ± 0.57	>0.05
*MDA, nmol/mgprot*	2.74 ± 0.57	2.15 ± 0.23	>0.05
*T-AOC, U/mL*	26.21 ± 1.36	32.90 ± 2.54*	<0.05

Data were analyzed with independent sample T-tests and expressed as mean ± SEM (n=6 per group).

* P < 0.05,

** P < 0.01 vs. HG group.

**Table 6:** Identified lipid-related differentially abundant metabolites.

Index	Metabolites	Formula	P-value	VIP	Trend	Metabolic pathways
*MEDN0383*	FFA(18:2)	C18H32O2	0.004	1.55	up	Linoleic acid metabolism
*MEDP1904*	LPE(18:2/0:0)	C23H44NO7P	0.007	1.54	up	glyceropgospholipid metabolism
*MEDP1221*	PC(O-16:0/O-1:0)	C25H54NO6P	0.001	1.54	down	glyceropgospholipid metabolism
*MW0012846*	[2-hydroxy-3-[(9Z,12Z)-octadeca-9,12-dienoyl]oxypropyl] 2-(trimethylazaniumyl)ethyl phosphate	C26H50NO7P	0.001	1.53	up	glyceropgospholipid metabolism

## References

[1] Wang Y, Li Q, Wang L, Liu Y, Yan T (2023). Effects of a High-Concentrate Diet on the Blood Parameters and Liver Transcriptome of Goats. Animals (Basel).

[2] Nasrollahi SM, Piadeh A, Kahyani H, Rahmati Andani A, Eyni B (2024). Effects of supplementing different feed additives to high-concentrate diets containing potassium carbonate on dairy cow performance. J Dairy Res.

[3] Ma N, Guo J, Li Z, Xu L, Zhang K, Xu T, Chang G, Loor JJ, Shen X (2024). Disturbances of Ruminal Microbiota and Liver Inflammation, Mediated by LPS and Histamine, in Dairy Cows Fed a High-Concentrate Diet. Animals (Basel).

[4] Ohtaki T, Ogata K, Kajikawa H, Sumiyoshi T, Asano S, Tsumagari S, Horikita T (2020). Effect of high-concentrate corn grain diet-induced elevated ruminal lipopolysaccharide levels on dairy cow liver function. J Vet Med Sci.

[5] Zhang Q, Jiang Y, Qin Y, Liu J, Xie Y, Zhang L, Li K, Wang X, Liu G (2024). Linoleic Acid Alleviates Lipopolysaccharide Induced Acute Liver Injury via Activation of Nrf2. Physiol Res.

[6] Zhao Y, Liu M, Xu J, Yu H, Li H, Bao L, Shi J, Wu K, Shan R, Zhang N, Hu X, Fu Y, Zhao C, Liang F, Han F (2025). Mitochondrial DNA contributes to dysbiosis-associated mastitis via activation of the cGAS-STING pathway. Int Immunopharmacol.

[7] Li L, Tang W, Zhao M, Gong B, Cao M, Li J (2021). Study on the regulation mechanism of lipopolysaccharide on oxidative stress and lipid metabolism of bovine mammary epithelial cells. Physiol Res.

[8] Li L, Cao Y, Xie Z, Zhang Y (2017). A High-Concentrate Diet Induced Milk Fat Decline via Glucagon-Mediated Activation of AMP-Activated Protein Kinase in Dairy Cows. Sci Rep.

[9] Huang Y, Wang Z, Ye B, Ma JH, Ji S, Sheng W, Ye S, Ou Y, Peng Y, Yang X, Chen J, Tang S (2023). Sodium butyrate ameliorates diabetic retinopathy in mice via the regulation of gut microbiota and related short-chain fatty acids. J Transl Med.

[10] Sun X, Luo S, Jiang C, Tang Y, Cao Z, Jia H, Xu Q, Zhao C, Loor JJ, Xu C (2020). Sodium butyrate reduces bovine mammary epithelial cell inflammatory responses induced by exogenous lipopolysaccharide, by inactivating NF-κB signaling. J Dairy Sci.

[11] Ali I, Li C, Kuang M, Shah AU, Shafiq M, Ahmad MA, Abdalmegeed D, Li L, Wang G (2022). Nrf2 Activation and NF-Kb & caspase/bax signaling inhibition by sodium butyrate alleviates LPS-induced cell injury in bovine mammary epithelial cells. Mol Immunol.

[12] Li L, Chen X, Yan S, Zhang Y (2024). Metabolomics Reveals the Mechanism by Which Sodium Butyrate Promotes the Liver Pentose Phosphate Pathway and Fatty Acid Synthesis in Lactating Goats. Animals (Basel).

[13] Ge L, Yu Y, Wen X, Xiao H, Liu K, Liu Z, Liu S, Li Q, Wang X, Deng Z, Hu Y (2023). Effects of dietary sodium butyrate on growth performance, immune function, and intestinal microflora of Chinese soft-shelled turtle (Pelodiscus sinensis). Front Cell Infect Microbiol.

[14] Dong J, Liu Y, Li S, Sun Z, Chen X, Wang D, Qin G, Zhang X, Aschalew ND, Wang T, Zhen Y (2023). The physiological dissimilarities of Holstein dairy cows with different milk yields. Vet Med Sci.

[15] Gross JJ, Grossen-Rösti L, Wall SK, Wellnitz O, Bruckmaier RM (2020). Metabolic status is associated with the recovery of milk somatic cell count and milk secretion after lipopolysaccharide-induced mastitis in dairy cows. J Dairy Sci.

[16] Shao D, Shen W, Miao Y, Gao Z, Pan M, Wei Q, Yan Z, Zhao X, Ma B (2023). Sulforaphane prevents LPS-induced inflammation by regulating the Nrf2-mediated autophagy pathway in goat mammary epithelial cells and a mouse model of mastitis. J Anim Sci Biotechnol.

[17] Meng M, Li X, Huo R, Ma N, Chang G, Shen X (2023). A high-concentrate diet induces mitochondrial dysfunction by activating the MAPK signaling pathway in the mammary gland of dairy cows. J Dairy Sci.

[18] Memon MA, Wang Y, Xu T (2019). Lipopolysaccharide induces oxidative stress by triggering MAPK and Nrf2 signalling pathways in mammary glands of dairy cows fed a high-concentrate diet. Microb Pathog.

[19] Aguilar EC, Da Silva JF, Navia-Pelaez JM (2018). Sodium butyrate modulates adipocyte expansion, adipogenesis, and insulin receptor signaling by upregulation of PPAR-γ in obese Apo E knockout mice. Nutrition.

[20] Li X, Wang C, Zhu J, Lin Q, Yu M, Wen J, Feng J, Hu C (2022). Sodium Butyrate Ameliorates Oxidative Stress-Induced Intestinal Epithelium Barrier Injury and Mitochondrial Damage through AMPK-Mitophagy Pathway. Oxid Med Cell Longev.

[21] Hou D, Li M, Li P, Chen B, Huang W, Guo H, Cao J, Zhao H (2023). Effects of sodium butyrate on growth performance, antioxidant status, inflammatory response and resistance to hypoxic stress in juvenile largemouth bass (Micropterus salmoides). Front Immunol.

[22] Memon MA, Dai H, Wang Y, Xu T, Aabdin ZU, Bilal MS, Chandra RA, Shen X (2019). Efficacy of sodium butyrate in alleviating mammary oxidative stress induced by sub-acute ruminal acidosis in lactating goats. Microb Pathog.

[23] Wu Y, Sun Y, Zhang R, He T, Huang G, Tian K, Liu J, Chen J, Dong G (2021). Sodium Butyrate More Effectively Mitigates the Negative Effects of High-Concentrate Diet in Dairy Cows than Sodium β-Hydroxybutyrate via Reducing Free Bacterial Cell Wall Components in Rumen Fluid and Plasma. Toxins (Basel).

[24] Zhao H, Bai H, Deng F, Zhong R, Liu L, Chen L, Zhang H (2022). Chemically protected sodium butyrate improves growth performance and early development and function of small intestine in broilers as one effective substitute for antibiotics. Antibiotics (Basel).

[25] Meng M, Li X, Huo R, Chang G, Shen X (2023). Effects of dietary disodium fumarate supplementation on muscle quality, chemical composition, oxidative stress and lipid metabolism of Hu sheep induced by high concentrate diet. Meat Sci.

[26] Zhang J, Bu L, Liu Y, Huo W, Xia C, Pei C, Liu Q (2023). Dietary supplementation of sodium butyrate enhances lactation performance by promoting nutrient digestion and mammary gland development in dairy cows. Anim Nutr.

[27] Dobrowolski A, Mirończuk AM (2020). The influence of transketolase on lipid biosynthesis in the yeast Yarrowia lipolytica. Microb Cell Fact.

[28] Ma N, Abaker JA, Wei G, Chen H, Shen X, Chang G (2022). A high-concentrate diet induces an inflammatory response and oxidative stress and depresses milk fat synthesis in the mammary gland of dairy cows. J Dairy Sci.

[29] Tzirkel-Hancock N, Sharabi L, Argov-Argaman N (2023). Milk fat globule size: Unraveling the intricate relationship between metabolism, homeostasis, and stress signaling. Biochimie.

[30] He W, Gao Z, Liu S, Tan L, Wu Y, Liu J, Zheng Z, Fan W, Luo Y, Chen Z, Song S (2023). G protein-coupled estrogen receptor activation by bisphenol-A disrupts lipid metabolism and induces ferroptosis in the liver. Environ Pollut.

[31] Jung TW, Lee SH, Kim HC, Bang JS, Abd El-Aty AM, Hacımüftüoğlu A, Shin YK, Jeong JH (2018). METRNL attenuates lipid-induced inflammation and insulin resistance via AMPK or PPARδ-dependent pathways in skeletal muscle of mice. Exp Mol Med.

[32] Cheng J, Zhang Y, Ge Y, Li W, Cao Y, Qu Y, Liu S, Guo Y, Fu S, Liu J (2020). Sodium butyrate promotes milk fat synthesis in bovine mammary epithelial cells via GPR41 and its downstream signalling pathways. Life Sci.

